# Beat to beat volumetric analysis in arrhythmia using real time CMR

**DOI:** 10.1186/1532-429X-17-S1-O37

**Published:** 2015-02-03

**Authors:** Francisco Contijoch, Hannah Rears, Kelly Rogers, Peter Kellman, Joseph H Gorman, Robert C Gorman, Walter R Witschey, Yuchi Han

**Affiliations:** 1 NIH, Bethesda, MD, USA; 2 University of Pennsylvania, Philadelphia, PA, USA

## Background

ECG-gated cardiac MRI is the gold standard for volumetric evaluation of patients, and clinically, ejection fraction is used as a surrogate for function. We hypothesized that the use of arrhythmia rejection in the presence of ectopic beats compromises the accuracy of hemodynamic measurements. Real-time MRI, coupled with ECG telemetry and semi-automated LV endocardial segmentation, can be used to identify multiple beat morphologies and derive global hemodynamic measurements for each beat.

## Methods

Short-axis golden angle radial bSSFP projections (8000 projections/slice) were reconstructed using Gadgetron (non-Cartesian, iterative SENSE) with 34 projections per frame, and slice volume was measured via segmentation of LV endocardial contour with ITK-SNAP. ECG was synchronously recorded with the acquisition, and each image frame was assigned a time point on the ECG (~22 seconds/slice). QRS detection was used to identify cardiac cycles, and similar beats (across slices) were grouped via RR-duration of the beat of interest as well as the previous (loading) beat. The use of the loading beat duration allowed for accurate grouping of premature ventricular contractions (PVCs). For each beat morphology, global volume was obtained by summation of slice volume curves allowing for hemodynamic evaluation via EDV, ESV, SV, and EF.

## Results

**Figure 1:**
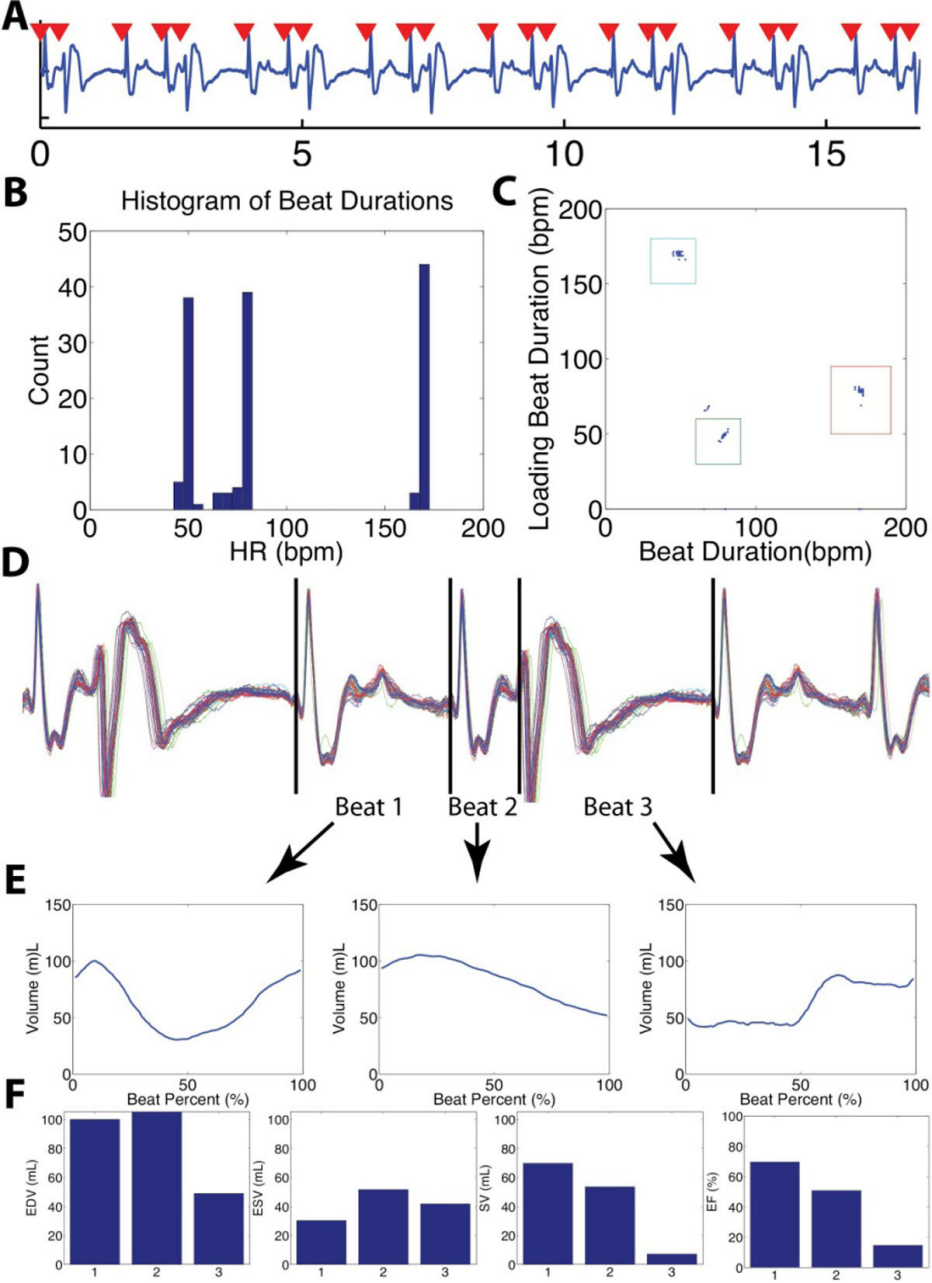
Results obtained from clinical patient with frequent PVC. A) ECG obtained with QRS detection results at one slice location. B) Histogram of RR-duration showing effect of PVCs. C) 2D plot of beat (x) and loading beat (y) durations allows for separation and identification of multiple beat morphologies. D) 3 beat morphologies were identified in this patient. The ECGs associated with the morphologies are shown. The first beat morphology is a normal depolarization which occurs after a prolonged diastolic period, the second beat morphology is a normal contraction which is interrupted during diastole by a PVC contraction, and the third morphology is a PVC. In this patient, this 3 beat pattern is consistently repeated. E) Global volume obtained by slice volume summation for each morphology. H) Volumetric measurements made for each beat show large variations depending on the morphology.

10 patients were imaged: 5 in sinus rhythm, 3 with PVCs with 19%, 24%, and 33% prevalence, 1 in regular bigeminy, and 1 in irregular bigeminy. All beats detected beats were assigned a morphology, excluding the first beat of each acquisition since the loading conditions of these beats were unknown. A single beat morphology was observed in patients in sinus rhythm while patients with rhythm disturbances demonstrated multiple beat morphologies with large variations in measured volumes and EF. Figure 1 shows real-time and composite hemodynamic measurements in a patient with frequent PVCs (33%). In this patient, the SV and EF varied from 7.2 - 69.9 mL and 14.7 - 69.7% respectively, depending on the beat morphology. This variation suggests that cardiac cine, even with perfect arrhythmia rejection, misses important aspects of ventricular function in the presence of arrhythmias.

## Conclusions

ECG-gated cardiac MRI is unable to capture the hemodynamics associated with arrhythmic events. As a result, values such as EF are acquisition - dependent (desired RR-duration determines arrhythmia rejection). By combining real-time volume measurements with ECG recordings, we categorized beat morphologies and provided a more comprehensive evaluation of ventricular function during arrhythmia.

## Funding

We would like to acknowledge the National Institutes of Health for support through grants: F31-HL120580, R00-HL108157, R01-EB014346, R01-HL103723, R01-HL63954, T32-HL007954, T32-EB009384.

